# Clinical characteristics, treatment patterns, and outcomes in adult patients with germline *BRCA1/2*-mutated, HER2-negative advanced breast cancer: a retrospective medical record review in the United States

**DOI:** 10.3389/fonc.2024.1341665

**Published:** 2024-05-16

**Authors:** Elias Obeid, Rohan C. Parikh, Elizabeth Esterberg, Bhakti Arondekar, Abigail Hitchens, Lillian Shahied Arruda, Alexander Niyazov, Kristen Whitaker

**Affiliations:** ^1^ Clinical Genetics, Fox Chase Cancer Center, Philadelphia, PA, United States; ^2^ Health Economics, RTI Health Solutions, Research Triangle Park, NC, United States; ^3^ Pfizer Inc., Collegeville, PA, United States; ^4^ Pfizer Inc., Department of US Oncology Medical Affairs, New York, NY, United States; ^5^ Pfizer Inc., Department of HTA, Value and Evidence, New York, NY, United States

**Keywords:** metastatic breast cancer, *BRCA1/2* gene mutations, poly (ADP-ribose) polymerase (PARP) inhibitors, human epidermal growth factor receptor 2 (HER2)-negative, hormone receptor-positive (HR+), triple-negative breast cancer (TNBC)

## Abstract

**Aim:**

To examine clinical characteristics, real-world treatment patterns, and health outcomes among patients with germline *BRCA1/2*-mutated, human epidermal growth factor receptor 2 (HER2)–negative advanced breast cancer (ABC).

**Methods:**

A retrospective analysis was conducted using medical records from patients with HER2-negative ABC with *BRCA1/2* mutation who received cytotoxic chemotherapy. Data were stratified into groups with triple-negative breast cancer (TNBC) or hormone receptor–positive (HR+)/HER2-negative diagnoses. Time-to-event outcomes (i.e., real-world progression-free survival [rwPFS] and overall survival [OS]) were calculated to summarize health outcomes.

**Results:**

When diagnosed with ABC, most patients were younger than 60 years (mean age = 57.3 years), were white (76.4%), and had a family history of *BRCA*-related cancer (71.5%). A total of 305 patient records were examined; 194 patients (63.6%) had advanced TNBC, and 111 patients (36.4%) had HR+/HER2-negative ABC. Chemotherapy was primarily used as first-line treatment for both subgroups, but the TNBC subgroup received poly (ADP-ribose) polymerase (PARP) inhibitors at triple the rate as a second-line treatment and double the rate as a third-line treatment compared with the HR+/HER2-negative subgroup. Two-year OS rates were similar between the TNBC (73.9%) and the HR+/HER2-negative subgroups (77.0%), and anemia, nausea, and neutropenia were the most commonly reported toxicities across all treatments.

**Conclusion:**

Clinicians should consider the use of targeted agents such as PARP inhibitors in earlier lines of therapy for ABC given the growing evidence that PARP inhibitors may improve PFS compared with chemotherapy while potentially offering a more manageable toxicity profile and improved quality of life.

## Introduction

In the United States (US), 1 in 8 women will be diagnosed with breast cancer during their lifetime (1), and among those, 1 in 3 will develop metastatic disease (2, 3). The overall 5-year survival rate for women with localized or regional nonmetastatic breast cancer is approximately 86%-99%, but for those with metastatic breast cancer, the 5-year survival rate drops substantially to 29% (4). Women with a genetic predisposition, such as those with a germline mutation in the *BRCA1/2* genes, have a significantly higher lifetime risk of developing breast cancer. Further, the cumulative lifetime risk of breast cancer for women who carry the *BRCA1/2* genes was estimated to be 72% for women who carry the *BRCA1* gene and 69% for women who carry the *BRCA2* gene, and women who had either *BRCA1* or *BRCA2* mutations carried greater risk for developing breast cancer if they had a family history (5). *BRCA1*/2 mutations have been shown to impact not only breast cancer risk but also breast cancer outcomes. Women with a *BRCA1* mutation and a breast cancer diagnosis have reduced time to progression and survival compared with *BRCA2* mutation carriers and noncarriers of *BRCA1/2* mutations (6). Additionally, treatment benefits from therapies such as adjuvant chemotherapy have been shown to be higher in patients without the *BRCA1/2* genetic mutations compared with patients with the mutations (7).

Treatment for advanced breast cancer (ABC) is often tailored based on clinical stage, tumor histology, biomarker expression, prior therapy, and patient performance status (8). Treatment in earlier stages of breast cancer may include surgical resection, breast- conserving surgery, mastectomy (simple or double mastectomy, and modified radical mastectomy), or radiation therapy in women with *BRCA1/2* mutation (9, 10). Historically, chemotherapy and/or endocrine therapy were the cornerstone of systemic treatment for patients with human epidermal growth factor receptor 2 (HER2)–negative ABC, but the treatment landscape is changing because of the recent introduction of therapies into the treatment paradigm. Recently, therapies such as poly (ADP-ribose) polymerase (PARP) inhibitors were approved in the US for the treatment of germline *BRCA1*/2-associated ABC (11–14). PARP inhibitors have been shown to improve the progression-free survival (PFS) compared with chemotherapy in patients with germline *BRCA1/2*-mutated, HER2-negative locally advanced and/or metastatic breast cancer (12, 15–17). Data from previous studies and the PRAEGNANT registry, which examines genetics and other molecular biomarkers in individuals with metastatic breast cancer, indicate that approximately 5% of patients with metastatic breast cancer carry germline *BRCA1/2* mutations (18, 19) and thus could potentially derive benefit from a PARP inhibitor. The optimal treatment sequencing in this population has not yet been established. Patient characteristics, prescribing patterns, diverse health care systems, and variable access to care in the clinical practice setting differ from controlled clinical trial environments, which can cause variation in real-world outcomes for patients with germline *BRCA1/2* mutations.

In this analysis, clinical characteristics, real-world treatment patterns, and health outcomes among patients with germline *BRCA1/2*-mutated, HER2-negative ABC in the US were evaluated in order to contextualize the results of relevant clinical trials and determine the extent of unmet need among these patients in the routine clinical-practice setting.

## Materials and methods

### Study design

This study was a noninterventional, retrospective medical record review of adult patients with HER2-negative ABC and germline *BRCA1/2* mutations who received cytotoxic chemotherapy. The RTI International Institutional Review Board determined that this study was not human subjects research (STUDY00020382), as the data were unidentifiable. ABC was defined as locally advanced breast cancer that was not amenable to curative radiation or surgery, or as metastatic disease. The sample was further stratified into two groups consisting of patients with advanced triple-negative breast cancer (TNBC) and patients with hormone receptor–positive (HR+)/HER2-negative ABC. Data describing patient and clinical characteristics, treatment patterns, and health outcomes were abstracted directly by eligible oncologists. A purposive, quasi-random sampling method was employed through the selection of medical records for patients whose last names began with a randomly generated letter. The inclusion criteria for oncologists, inclusion criteria for medical records, and exclusion criteria for medical records are presented in **Table 1**. Medical record abstraction occurred from July 2019 to May 2020, which allowed for extraction of follow-up data (when available) until May 2020.

**Table 1:** Inclusion/Exclusion criteria.

Inclusion criteria for physician data abstractors
▪ Practicing oncologists or hematologist-oncologists ▪ Treated at least 1 patient with ABC with a *BRCA* mutation in the 24 months before data abstraction ▪ Acted as main decision maker regarding ABC treatment or follow-up
Inclusion criteria for medical records
▪ Female or male patients aged > 18 years with germline *BRCA1/2*-mutated, HER2-negative ABC (i.e., local ABC that was not amenable to curative radiation or surgical cure or metastatic breast cancer), living or deceased at the time of data abstraction ▪ Initiated > 1 cytotoxic chemotherapy (single agent or combination) for advanced disease (any line of therapy) from January 1, 2013, to April 30, 2018^a^ ▪ Use of nonchemotherapy-based treatments as first-line treatment for advanced disease was permitted
Exclusion criteria for medical records
▪ Patients with evidence of other malignant neoplasms except: ▪ Nonmelanoma skin cancer ▪ Carcinoma in situ ▪ *BRCA* mutation carrier with a prior history of ovarian or fallopian tube cancer that was cured before initial breast cancer diagnosis ▪ Prior nonbreast cancer diagnosis from which the patient had been disease-free for > 5 years) ▪ Patients who participated in a clinical trial related to treatment of ABC

ABC, advanced breast cancer; *BRCA*, germline breast cancer gene; HER2, human epidermal growth factor receptor 2.

a Limiting the latest index date to April 2018 allowed a follow-up opportunity of >12 months (data collection started in July 2019) and therefore mature data.

### Demographics and clinical characteristics

Demographic characteristics, including primary medical specialty, primary practice setting, and geographic region of practice, were collected for the recruited oncologists. For eligible patients, demographic data, as well as baseline clinical characteristics related to their initial breast cancer diagnosis and the diagnosis of ABC, were collected from medical records. Patient demographic data included sex, race, and family history of a *BRCA*-related cancer. Baseline clinical characteristics included age and Eastern Cooperative Oncology Group (ECOG) performance status at ABC diagnosis.

### Treatment patterns

**Figure 1:**
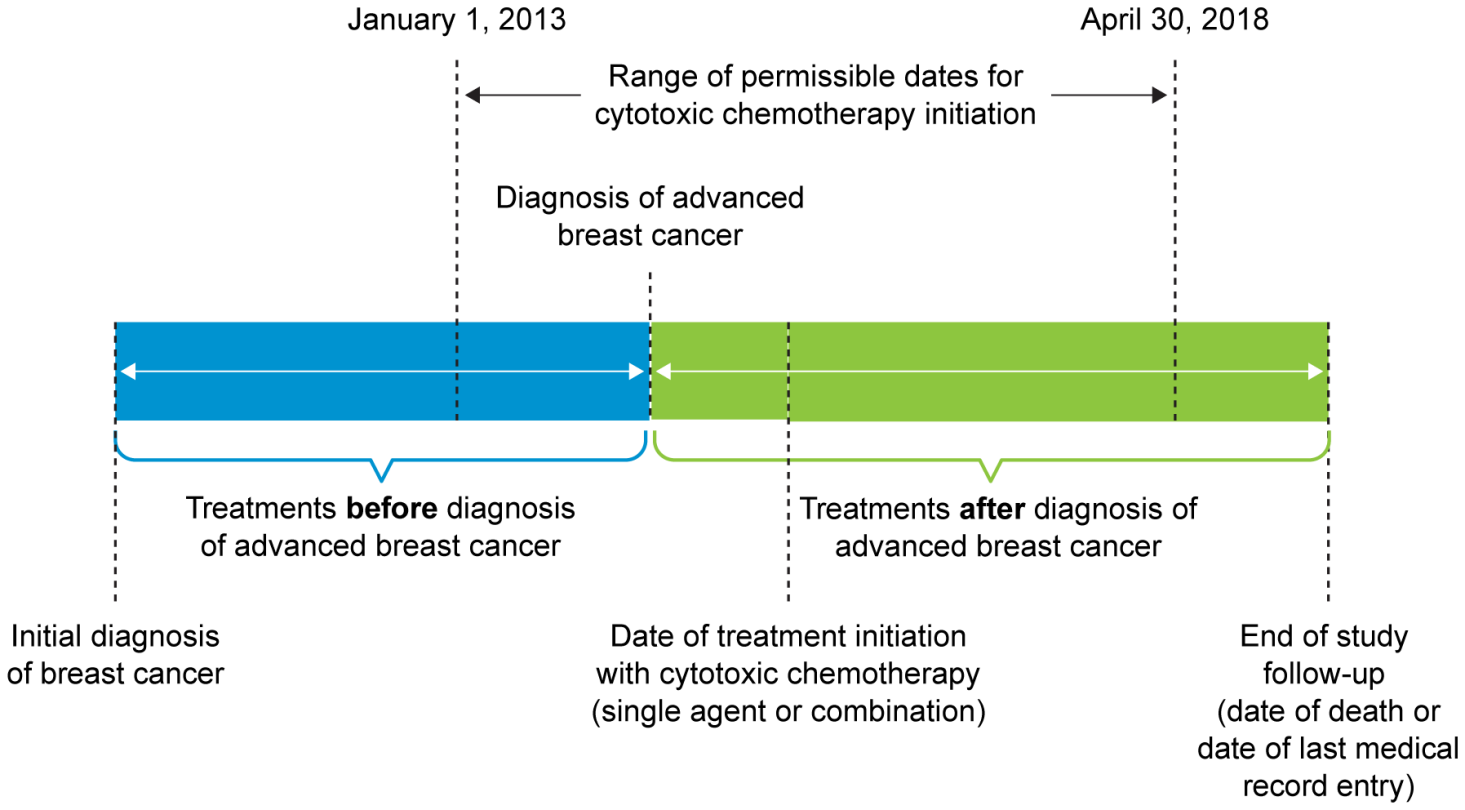
Treatment Pattern Time Period. Patients who were initially diagnosed with advanced breast cancer or were initially diagnosed at an earlier stage and experienced progression to advanced breast cancer were included in the study.

Data measures related to the treatment of breast cancer were collected before and after diagnosis of ABC (**Figure 1**). Treatment choices before the diagnosis of ABC included neoadjuvant treatment(s), surgery, adjuvant chemotherapy, adjuvant targeted therapy/biologics, adjuvant hormonal therapy, and radiation therapy. Types of treatment approaches, along with total number of systemic lines, collected after diagnosis of ABC included surgery, chemotherapy, targeted therapy/biologics, hormonal therapy, and radiation therapy.

### Health outcomes

Health outcomes included time-to-event estimates (i.e., real-world PFS [rwPFS], overall survival [OS]) and prespecified treatment-related toxicities. Dates of progression and death (i.e., whether the patient died during the follow-up period and whether the death was related to breast cancer) were documented. Time-to-event estimates were calculated from the date of ABC diagnosis and the start of each treatment line (received after ABC diagnosis). Several treatment-related hematological (i.e., anemia, neutropenia, thrombocytopenia) and nonhematologic toxicities (i.e., hand-foot syndrome [or palmar-plantar erythrodysesthesia], nausea, vomiting) commonly observed in clinical trials and real-world studies (10, 20) were documented for each treatment line received after diagnosis of ABC.

### Statistical analysis

Measures were summarized descriptively through tabular and graphical display of mean values, medians, ranges, and standard deviations for continuous variables of interest and frequency distributions for categorical variables. Time-to-event outcomes (i.e., rwPFS, OS) were calculated using Kaplan-Meier methods. All analyses were conducted using SAS (version 9.4 or higher) statistical software. This study was descriptive, with the primary goal of retrospectively summarizing existing treatment patterns and patient outcomes.

## Results

### Demographics and clinical characteristics

A total of 97 oncologists provided abstraction of 305 medical records of adult patients with germline *BRCA1/2*-mutated, HER2-negative ABC. The majority were medical oncologists (62.9%) and practiced in a private hospital or clinic (48.5%) (**Supplementary Table 1**). The geographic locations were approximately evenly distributed among the Southern (30.9%), Northeastern (26.8%), Midwestern (21.7%), and Western US (20.6%).

Patient demographics and clinical characteristics are presented in **Table 2**. Of the 305 included medical records, 194 (63.6%) patients had advanced TNBC and 111 (36.4%) patients had HR+/HER2-negative ABC. The median age at ABC diagnosis for the overall patient population with germline *BRCA1/2*-mutated, HER2-negative ABC was 57.3 (range, 33.3-79.8) years. Similar median ages at ABC diagnosis were found for the TNBC (57.2 years) and HR+/HER2-negative (58.2 years) subgroups. There was one male patient in the TNBC subgroup. The overall group was mostly white (76.4%); this composition decreased for the TNBC subgroup (71.7%) and increased for the HR+/HER2-negative subgroup (84.7%). The percentages of patients of Ashkenazi Jewish ancestry were similar across the overall group (11.5%), the TNBC subgroup (12.9%), and the HR+/HER2-negative subgroup (9.0%). The percentages of patients with no known family history of a *BRCA*-related cancer were 24.6%, 27.8%, and 18.9% for the overall group, the TNBC subgroup, and the HR+/HER2-negative subgroup, respectively. The percentage of patients with an ECOG performance status of 0 or 1 at ABC diagnosis was 74.6% for the overall group, 70.2% for the TNBC subgroup, and 83.2% for the HR+/HER2-negative subgroup.

**Table 2:** Demographics and clinical characteristics in patients with germline *BRCA1/2*-mutated HER2-negative advanced breast cancer.

	Overall (N = 305)	TNBC (n = 194)	HR+/HER2− (n = 111)
Age at ABC diagnosis, median, y	57.3	57.2	58.2
Sex, n (%)			
Female	304 (99.7)	193 (99.5)	111 (100.0)
Male	1 (0.3)	1 (0.5)	0 (0.0)
Race, n (%)^a^			
White	233 (76.4)	139 (71.7)	94 (84.7)
Black/African American	57 (18.7)	43 (22.2)	14 (12.6)
Asian, Native Hawaiian, or other Pacific Islander	15 (4.9)	12 (6.2)	3 (2.7)
Other	1 (0.3)	1 (0.5)	0 (0.0)
Ashkenazi Jewish descent, n (%)	35 (11.5)	25 (12.9)	10 (9.0)
Known family history of a *BRCA*-related cancer, n (%)	218 (71.5)	135 (69.6)	83 (74.8)
No known family history of a *BRCA*-related cancer, n (%)	75 (24.6)	54 (27.8)	21 (18.9)
Unsure of family history of a *BRCA*-related cancer, n (%)	12 (3.9)	5 (2.6)	7 (6.3)
ECOG performance status at ABC diagnosis, n (%)	276 (90.5)	181 (93.3)	95 (85.6)
0 or 1, n (%)	206 (74.6)	127 (70.2)	79 (83.2)

ABC, advanced breast cancer; *BRCA*, germline breast cancer gene; ECOG, Eastern Cooperative Oncology Group; HER2, human epidermal growth factor receptor 2; HR, hormone receptor; TNBC, triple-negative breast cancer.

a 1 patient was reported as White and Asian, Native Hawaiian, or other Pacific Islander.

### Treatment patterns

**Figure 2:**
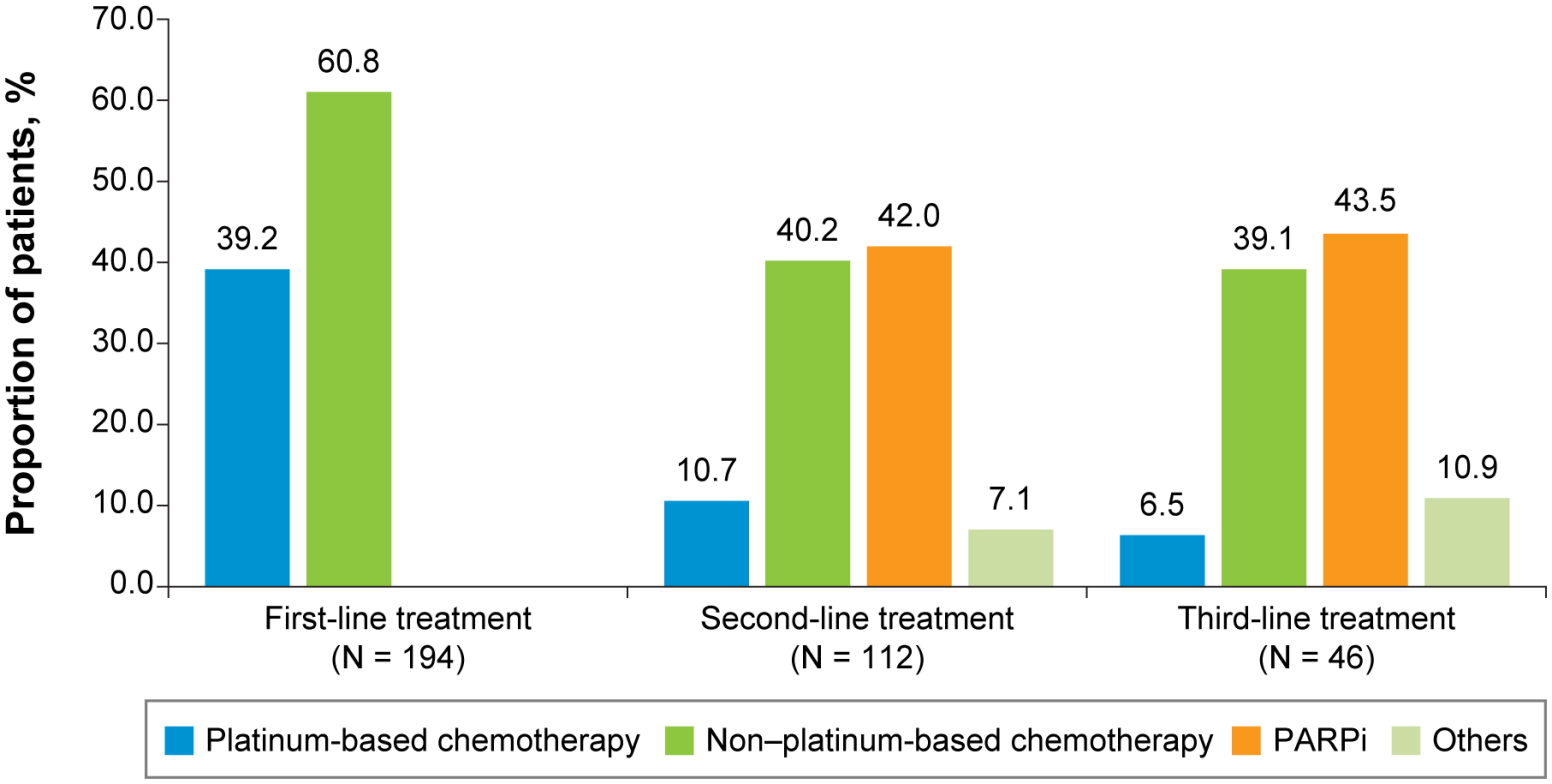
First-, Second-, and Third-Line Treatments Among Patients With Advanced Triple-Negative Breast Cancer. PARPi, poly (ADP-ribose) polymerase inhibitors.

**Figure 3:**
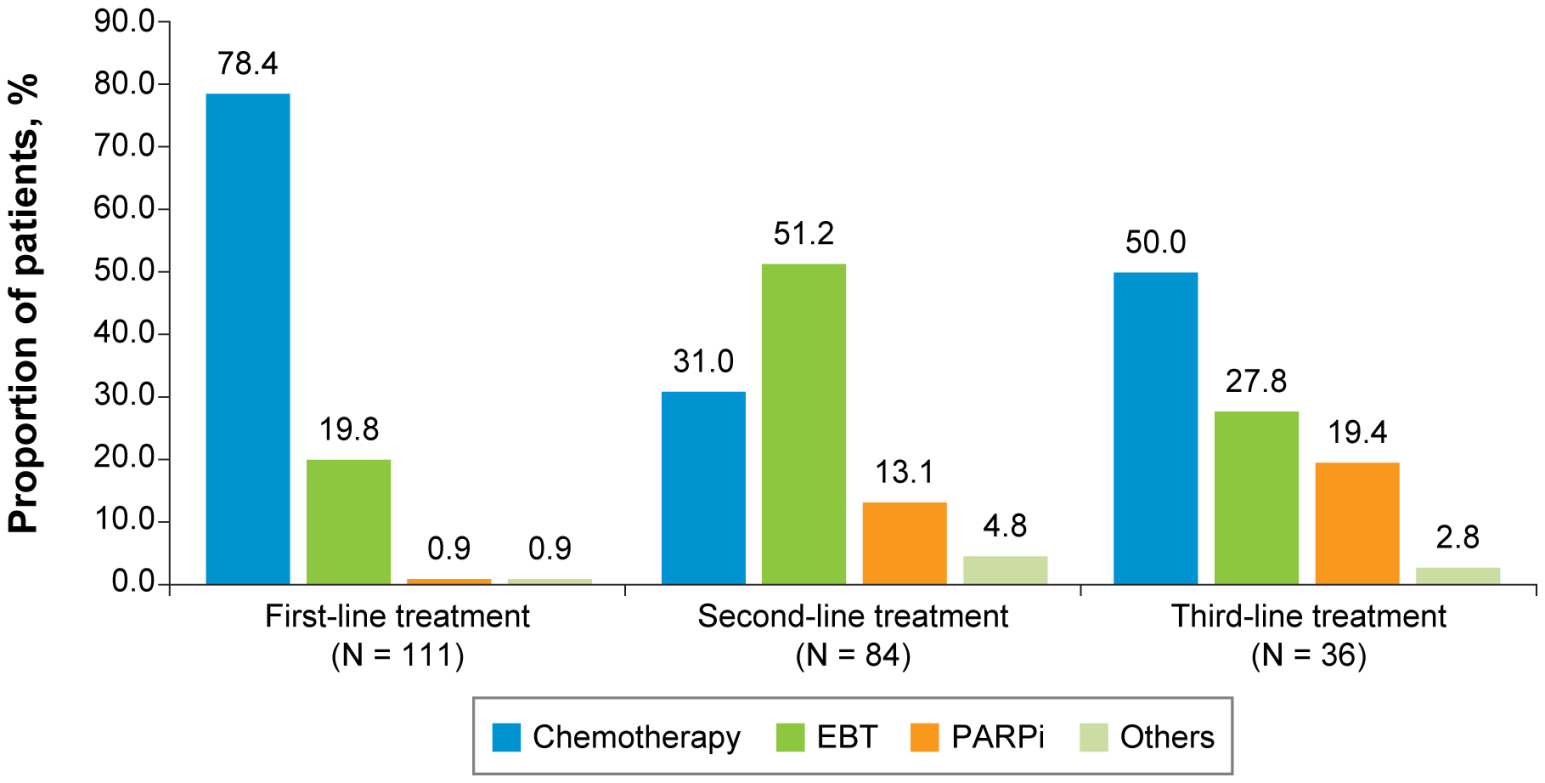
First-, Second-, and Third-Line Treatments Among Patients With HR+/HER2-Negative ABC. ABC, advanced breast cancer; EBT, endocrine-based therapy; HER2, human epidermal growth factor receptor 2; HR, hormone receptor; PARPi, poly (ADP-ribose) polymerase inhibitors.

For both the TNBC (**Figure 2**) and HR+/HER2-negative (**Figure 3**) subgroups, first-line treatments were similar, with chemotherapy being the most commonly used treatment, but differences in treatments between these subgroups emerged in the second-line setting and beyond. First-line treatment for the TNBC subgroup was composed of mostly nonplatinum-based chemotherapy (60.8%) and platinum-based chemotherapy (39.2%). In second-line treatment, the TNBC subgroup had nearly equivalent rates of nonplatinum-based chemotherapy (40.2%) and PARP inhibitors (42.0%). Third-line treatment in the TNBC subgroup again had similar rates for nonplatinum-based chemotherapy (39.1%) and PARP inhibitors (43.5%). In the HR+/HER2-negative subgroup, first-line treatment consisted of chemotherapy for 78.4% of patients and endocrine-based therapy (EBT) for 19.8% of patients. In second-line treatment, most of the HR+/HER2-negative subgroup received EBT (51.2%), with lower percentages of chemotherapy (31.0%) and PARP inhibitors (13.1%). In third-line treatment for the HR+/HER2-negative subgroup, chemotherapy again became the dominant treatment (50.0%), with decreased use of EBT (27.8%) and increased use of PARP inhibitors (19.4%).

### Efficacy and safety outcomes

During the first-, second-, and third-line treatments, rwPFS was assessed for the TNBC and HR+/HER2-negative ABC subgroups (**Table 3**). Among patients with TNBC, the median PFS for first-line treatment (n = 190) was 11.6 months (95% confidence interval [CI], 9.9-13.6); for second-line treatment (n = 64), the median PFS was 9.0 months (95% CI, 6.2-11.4); and for third-line treatment (n = 26), the median PFS was 5.9 months (95% CI, 3.5-10.9). Among patients who were HR+/HER2 negative, the median PFS for first-line treatment (n = 110) was 12.1 months (95% CI, 9.1-14.3); for second-line treatment (n = 68), the median PFS was 14.2 months (95% CI, 10.7-29.5); and for third-line treatment (n = 29), the median PFS was 6.5 months (95% CI, 4.4-9.2). Overall survival from index chemotherapy was assessed for the TNBC and HR+/HER2-negative ABC subgroups (**Table 4**). Patients in the TNBC subgroup had a 2-year OS rate of 73.9%, which decreased to 53.1% at 5 years. The median OS was not estimable during the study follow-up. Patients in the HR+/HER2-negative subgroup had a 2-year OS rate of 77.0%, which decreased to 44.2% at 5 years, and a median OS of 45.9 months.

**Table 3:** Progression-free survival during first-, second-, and third-line treatment.

	Advanced TNBC	Advanced HR+/HER2−
First-line treatment		
n^a^	190	110
PFS, mo^b^		
Progressed or died, n (%)	117 (61.6)	63 (57.3)
Time from initiation of treatment (Kaplan-Meier estimate)		
Mean (SE)	16.0 (0.9)	13.1 (0.8)
Median	11.6	12.1
95% CI	9.9-13.6	9.1-14.3
Censored, n (%)	73 (38.4)	47 (42.7)
Second-line treatment		
n^a^	64	68
PFS, mo^b^		
Progressed or died, n (%)	35 (54.7)	37 (54.4)
Time from initiation of treatment (Kaplan-Meier estimate)		
Mean (SE)	10.1 (0.9)	28.2 (3.4)
Median	9.0	14.2
95% CI	6.2-11.4	10.7-29.5
Censored, n (%)	29 (45.3)	31 (45.6)
Third-line treatment		
n^a^	26	29
PFS, mo^b^		
Progressed or died, n (%)	15 (57.7)	24 (82.8)
Time from initiation of treatment (Kaplan-Meier estimate)		
Mean (SE)	7.1 (0.9)	6.8 (0.6)
Median	5.9	6.5
95% CI	3.5-10.9	4.4-9.2
Censored, n (%)	11 (42.3)	5 (17.2)

ABC, advanced breast cancer; HER2, human epidermal growth factor receptor 2; HR, hormone receptor; PFS, progression-free survival; SE, standard error; TNBC, triple-negative breast cancer.

a Number of patients who received first-line treatment and for whom progression was assessed. Progression was not assessed among patients who received poly (ADP-ribose) polymerase (PARP) inhibitors.

b Physician-assessed disease progression and death were considered “progression events” for PFS calculation. Progression events were documented as the reason for stopping the treatment, best response to treatment, during the treatment, or between the end of treatment and start of next treatment.

**Table 4:** Overall survival and mortality among patients with germline *BRCA1/2*-mutated, HER2-negative advanced breast cancer.

	TNBC (n = 194)	HR+/HER2− (n= 111)
All-cause from index chemotherapy, mo		
Kaplan-Meier estimate		
Mean (SE)	36.0 (1.5)	33.7 (1.8)
Median	NE	45.9
95% CI	32.5-NE	29.1-NE
Censored, n (%)	134 (69.1)	79 (71.2)
Survival rate, % (SE), mo		
12	83.9 (2.6)	88.2 (3.1)
24	73.9 (3.4)	77.0 (4.4)
36	56.9 (5.1)	51.6 (8.0)
48	56.9 (5.1)	44.2 (9.7)
60	53.1 (6.0)	44.2 (9.7)

*BRCA*, germline breast cancer gene; CI, confidence interval; HER2, human epidermal growth factor receptor 2; HR, hormone receptor; NE, not estimable; SE, standard error; TNBC, triple-negative breast cancer.

The prespecified treatment-related hematological toxicities and evidence of hand-foot syndrome, nausea, and vomiting during the first-, second-, and third-line treatments were documented for the TNBC and HR+/HER2-negative ABC subgroups (**Table 5**). For first-line treatment, the most commonly experienced toxicities for both the TNBC (n = 190) and HR+/HER2-negative (n = 110) subgroups were nausea (41.1% and 27.3%, respectively) and anemia (26.3% and 20.9%, respectively). Toxicities were not documented for 24.7% of the patients in the TNBC subgroup and 44.6% of patients in the HR+/HER2-negative subgroup in the first-line setting. For second-line treatment, the TNBC subgroup (n = 64) documented anemia (32.8%) and the HR+/HER2-negative subgroup (n = 68) documented neutropenia (17.7%) as the most commonly experienced toxicities. Toxicities were not documented for 32.8% of the TNBC subgroup and 51.5% of the HR+/HER2-negative subgroup in the second-line setting. For third-line treatment, nearly half (44.8%) of the 29 patients in the HR+/HER2-negative subgroup did not document toxicities, compared with 15.4% of patients who did not document toxicities in the TNBC subgroup (n = 26). However, the small sample sizes for third-line treatment warrant caution in interpretation of the toxicities experienced.

**Table 5:** Toxicities during first-, second-, and third-line treatment.

Toxicity, n (%)	Advanced TNBC	Advanced HR+/HER2−
First-line treatment		
**Patients initiating treatment**	(n = 190)	(n = 110)
Anemia	50 (26.3)	23 (20.9)
Neutropenia	30 (15.8)	12 (10.9)
Thrombocytopenia	23 (12.1)	9 (8.2)
Hand-foot syndrome	12 (6.3)	3 (2.7)
Nausea	78 (41.1)	30 (27.3)
Vomiting	40 (21.1)	10 (9.1)
No toxicities/syndrome experienced	47 (24.7)	49 (44.6)
Second-line treatment		
**Patients initiating treatment**	(n = 64)	(n = 68)
Anemia	21 (32.8)	6 (8.8)
Neutropenia	8 (12.5)	12 (17.7)
Thrombocytopenia	9 (14.1)	2 (2.9)
Hand-foot syndrome	2 (3.1)	2 (2.9)
Nausea	15 (23.4)	5 (7.4)
Vomiting	1 (1.6)	0 (0.0)
No toxicities/syndrome experienced	21 (32.8)	35 (51.5)
Third-line treatment		
**Patients initiating treatment**	(n = 26)	(n = 29)
Anemia	7 (26.9)	5 (17.2)
Neutropenia	3 (11.5)	3 (10.3)
Thrombocytopenia	2 (7.7)	2 (6.9)
Hand-foot syndrome	4 (15.4)	0 (0.0)
Nausea	3 (11.5)	5 (17.2)
Vomiting	2 (7.7)	0 (0.0)
No toxicities/syndrome experienced	4 (15.4)	13 (44.8)

HER2, human epidermal growth factor receptor 2; HR, hormone receptor; TNBC, triple-negative breast cancer.

Categories are not mutually exclusive. A patient may have had more than one toxicity or may be missing data; thus, the columns do not sum to 100%.

## Discussion

The current study involved a relatively large review of medical records that described the clinical characteristics, treatment patterns, rwPFS, and OS of adult patients with HER2-negative ABC with germline *BRCA1/2* mutation who had received cytotoxic chemotherapy. At the time of ABC diagnosis, most patients in this sample were younger than 60 years (mean age = 57.3 years), were white (76.4%), and had a family history of *BRCA*-related cancer (71.5%). Nearly 25% of the patients who were germline *BRCA1/2* mutation carriers in this study had no known family history of *BRCA*-related cancers, thus emphasizing the importance of not limiting *BRCA1/2* genetic testing to those who have family history. Two-year OS rates were similar between the advanced TNBC subgroup (73.9%) and the HR+/HER2-negative subgroup (77.0%). Anemia, nausea, and neutropenia were among the most commonly documented toxicities across all treatment lines, although some patients had no documentation of experiencing the prespecified toxicities. Because of our selection criteria, chemotherapy was primarily used as first-line treatment for both TNBC and HR+/HER2-negative subgroups. However, compared with the HR+/HER2-negative subgroup, the TNBC subgroup received PARP inhibitors at triple the rate in the second-line setting and double the rate in the third-line setting. The introduction of PARP inhibitors for second-line treatment of TNBC could be linked to the increased proportion of patients who did not experience toxicities, from 24.7% in the first line to 32.8% in the second line. The relatively higher use of PARP inhibitors in the TNBC subgroup compared with the HR+/HER2-negative subgroup likely reflects that there are limited nonchemotherapy treatment options for patients with triple-negative disease, whereas patients with HR+/HER2-negative ABC have other nonchemotherapy-targeted treatment options, such as CDK4/6 inhibitors, *PIK3CA* inhibitors, and mTOR inhibitors, combined with endocrine therapy that can be utilized in the second and third lines.

Recent clinical trials have demonstrated that the use of PARP inhibitors—a nonchemotherapy-targeted treatment option—has significantly improved clinical and patient-reported outcomes with a manageable side effect profile in patients with HER2-negative ABC carrying a germline *BRCA1/2* mutation (12, 15, 16). OlympiAD, a phase 3 clinical trial of patients with germline *BRCA1/2*-mutated, HER2-negative ABC, showed that a significantly longer median PFS was observed in patients who received olaparib (7.0 months; n = 205 patients), a PARP inhibitor treatment, compared with patients who received single-agent chemotherapy (either capecitabine, eribulin, or vinorelbine), for whom PFS was 4.2 months (n = 97 patients, *P* < 0.001) (12). Similarly, EMBRACA, a phase 3 clinical trial of patients with germline *BRCA1/2*-mutated, HER2-negative ABC, demonstrated significantly longer median PFS in patients who received talazoparib (8.6 months; n = 287 patients), a PARP inhibitor treatment, compared with patients who received 1 of 4 physician’s choice standard chemotherapy options, for whom PFS was 5.6 months (n = 144, *P* < 0.001) (15). A safety analysis using the EMBRACA trial data evaluated the safety profile of talazoparib in 286 patients compared with chemotherapy in 126 patients with germline *BRCA1/2*, HER2-negative mutation and ABC (16). While anemia and nausea were commonly experienced adverse events, talazoparib participants experiencing these events had more favorable patient-reported outcomes compared with participants on chemotherapy.

Therefore, an accumulating body of evidence suggests patients who receive PARP inhibitors have better PFS, more favorably reported outcomes, and fewer instances of discontinuation secondary to side effects compared with patients treated with chemotherapy. To date, the sequencing of treatment options, particularly when to introduce PARP inhibitors into the treatment of patients with HER2-negative germline *BRCA1/2* ABC, has yet to be explored. Targeted therapy with PARP inhibitors may need to be considered in earlier lines of treatment in patients with ABC. This consideration is especially relevant among patients with TNBC whose alternative treatment options will consist of chemotherapy alone or chemotherapy combined with immunotherapy, which are associated with significant toxicities and limited improvement in breast cancer outcomes (i.e., PFS and/or OS).

### Study limitations

This study represents the first dataset to examine real-world treatment patterns and outcomes exclusively among HER2-negative patients with germline *BRCA1/2*-mutated ABC and provides valuable findings related to how targeted therapies, such as PARP inhibitors, are being incorporated into real-world practice in this select patient population. However, given that data abstraction occurred between July 2019 and May 2020 (maximum follow-up until May 2020), the incidence of PARP inhibitor therapy may have changed, and PARP inhibitors might be prescribed more often. A limitation of this study is that patients selected for study inclusion represented a purposive sample of medical records obtained from oncologists who were willing to participate in the study. Therefore, study findings may not be generalizable to the overall population of patients with germline *BRCA1/2*-mutated ABC. To minimize this limitation and improve generalizability of findings, oncologists were recruited from a variety of geographic regions and practice types. Another limitation was that data were limited to medical records to which oncologists had access. Information on health care services received outside the physician’s care setting that was not recorded in the medical record was unavailable for this study (e.g., treatment for adverse events received through care rather than the abstracting physician specialist). Accordingly, some adverse events may not have been included because they were reported elsewhere and not collected in the medical record. Also, while not all known treatment-related toxicities were evaluated in this study, toxicities commonly observed in clinical trials and real-world studies were assessed. Lastly, to homogenize the sample, patients in this study were required to have initiated at least one cytotoxic chemotherapy for ABC, which may have resulted in selection bias and could be the reason for relatively lower utilization of endocrine or endocrine-based therapies and PARP inhibitors observed in our study population.

### Key findings

Given the growing evidence that PARP inhibitors may improve PFS better than chemotherapy while potentially offering a more manageable toxicity profile and improved quality of life, clinicians should consider the use of targeted agents such as PARP inhibitors in earlier lines of therapy for ABC. Further prospective trials examining the sequencing of targeted agents in ABC are needed. Ultimately, the results of this study may contextualize the results of ongoing clinical trials, inform routine clinical-practice, and improve patient lives.

## Data availability statement

The datasets presented in this article are not readily available because they are not public. Requests to access the datasets should be directed to rparikh@rti.org.

## Ethics statement

Ethical approval was not required for the study involving humans in accordance with the local legislation and institutional requirements. Written informed consent to participate in this study was not required from the participants or the participants’ legal guardians/next of kin in accordance with the national legislation and the institutional requirements.

## Author contributions

EO: Investigation, Supervision, Visualization, Writing – review & editing. RCP: Conceptualization, Data curation, Formal analysis, Investigation, Methodology, Project administration, Resources, Software, Supervision, Visualization, Writing – original draft. EE: Formal analysis, Investigation, Methodology, Software, Supervision, Writing – review & editing. BA: Conceptualization, Funding acquisition, Investigation, Methodology, Writing – review & editing. AH: Formal analysis, Investigation, Methodology, Software, Writing – review & editing. LSA: Funding acquisition, Investigation, Supervision, Writing – original draft, Writing – review & editing. AN: Conceptualization, Data curation, Formal analysis, Funding acquisition, Investigation, Methodology, Project administration, Resources, Supervision, Visualization, Writing – original draft, Writing – review & editing. KW: Investigation, Supervision, Visualization, Writing – review & editing.

## References

[1] The American Cancer Society medical and editorial content team. How common is breast cancer? The American Cancer Society (2021). Accessed on May 23, 2021. Available at: https://www.cancer.org/cancer/breast-cancer/about/how-common-is-breast-cancer.html.

[2] O’Shaughnessy J. Extending survival with chemotherapy in metastatic breast cancer. Oncologist. (2005) 10 Suppl 3:20–9. doi: 10.1634/theoncologist.10-90003-20 16368868

[3] Redig AJ McAllister SS. Breast cancer as a systemic disease: a view of metastasis. J Intern Med. (2013) 274:113–26. doi: 10.1111/joim.12084 PMC3711134 23844915

[4] National Cancer Institute Surveillance, Epidemiology, and End Results Program. Cancer stat facts: female breast cancer. Accessed on May 3, 2021. Available at: https://seer.cancer.gov/statfacts/html/breast.html.

[5] Kuchenbaecker KB Hopper JL Barnes DR Phillips KA Mooij TM Roos-Blom MJ. Risks of breast, ovarian, and contralateral breast cancer for BRCA1 and BRCA2 mutation carriers. JAMA. (2017) 317:2402–16. doi: 10.1001/jama.2017.7112 28632866

[6] Bayraktar S Gutierrez-Barrera AM Lin H Elsayegh N Tasbas T Litton JK. Outcome of metastatic breast cancer in selected women with or without deleterious BRCA mutations. Clin Exp Metastasis. (2013) 30:631–42. doi: 10.1007/s10585-013-9567-8 PMC3857726 23370825

[7] Shah PD Patil S Dickler MN Offit K Hudis CA Robson ME. Twenty-one-gene recurrence score assay in BRCA-associated versus sporadic breast cancers: differences based on germline mutation status. Cancer. (2016) 122:1178–84. doi: 10.1002/cncr.29903 26859126

[8] Turashvili G Brogi E. Tumor heterogeneity in breast cancer. Front Med (Lausanne). (2017) 4:227. doi: 10.3389/fmed.2017.00227 29276709 PMC5727049

[9] Evron E Ben-David AM Goldberg H Fried G Kaufman B Catane R. Prophylactic irradiation to the contralateral breast for BRCA mutation carriers with early-stage breast cancer. Ann Oncol. (2019) 30:412–7. doi: 10.1093/annonc/mdy515 30475942

[10] Society AC. Treating breast cancer 2021. Available online at: https://www.cancer.org/cancer/breast-cancer/treatment.html.

[11] US Food and Drug Administration. Talazoparib prescribing information. Pfizer Labs. (2018), Accessed on April 22, 2021.

[12] Robson M Im SA Senkus E Xu B Domchek SM Masuda N. Olaparib for metastatic breast cancer in patients with a germline BRCA mutation. N Engl J Med. (2017) 377:523–33. doi: 10.1056/NEJMoa1706450 28578601

[13] Lord CJ Tutt AN Ashworth A. Synthetic lethality and cancer therapy: lessons learned from the development of PARP inhibitors. Annu Rev Med. (2015) 66:455–70. doi: 10.1146/annurev-med-050913-022545 25341009

[14] Pommier Y O’Connor MJ de Bono J. Laying a trap to kill cancer cells: PARP inhibitors and their mechanisms of action. Sci Transl Med. (2016) 8:362ps17. doi: 10.1126/scitranslmed.aaf9246 27797957

[15] Litton JK Rugo HS Ettl J Hurvitz SA Goncalves A Lee KH. Talazoparib in patients with advanced breast cancer and a germline BRCA mutation. N Engl J Med. (2018) 379:753–63. doi: 10.1056/NEJMoa1802905 PMC10600918 30110579

[16] Hurvitz SA Gonçalves A Rugo HS Lee KH Fehrenbacher L Mina LA. Talazoparib in patients with a germline BRCA-mutated advanced breast cancer: Detailed safety analyses from the phase III EMBRACA trial. Oncologist. (2020) 25:e439–e50. doi: 10.1634/theoncologist.2019-0493 PMC7066700 32162822

[17] National Comprehensive Cancer Network. NCCN clinical practice guidelines in oncology Version 6. 2020. National Comprehensive Cancer Network (3025 Chemical Road, Suite 100, Plymouth Meeting, PA 19462), Accessed on October 14, 2020. https://www.nccn.org/professionals/physician_gls/pdf/breast.pdf.

[18] Armstrong N Ryder S Forbes C Chalker A Ross J Quek R. An international systematic review (SR) of breast cancer (BC) BRCA mutation (BRCAm) prevalence. Ann Oncol. (2018) 29:viii97–viii8. doi: 10.1093/annonc/mdy272.297

[19] Fasching PA Hu C Hart S Hartkopf AD Taran FA Janni W. Germline BRCA1and BRCA2 mutations in patients with HER2-negative metastatic breast cancer (mBC) treated with first-line chemotherapy: Data from the German PRAEGNANT registry. J Clin Oncol. (2019) 37:1048. doi: 10.1200/JCO.2019.37.15_suppl.1048

[20] Anders CK LeBoeuf NR Bashoura L Faiz SA Shariff AI Thomas A. What’s the price? Toxicities of targeted therapies in breast cancer care. Am Soc Clin Oncol Educ Book. (2020) 40:55–70. doi: 10.1200/EDBK_279465 32421449

