# Supplementary material for: Clinical characteristics, treatment patterns, and outcomes in adult patients with germline BRCA1/2-mutated, HER2-negative advanced breast cancer: a retrospective medical record review in the United States
Source: Front Oncol. 2024 May 16;14:1341665. doi: 10.3389/fonc.2024.1341665 (PMC11137205; doi:10.3389/fonc.2024.1341665)
Supplement: Supplementary file 1 [file Table_1.docx]

# Supplementary Material

Table S-1. Physician Characteristics

| Physician Characteristic | Physicians (N = 97), | |
| --- | --- | --- |
|  | n | % |
| Primary medical specialty |  |  |
| Medical oncologist | 61 | 62.9 |
| Hematologist-oncologist | 36 | 37.1 |
| Primary practice setting |  |  |
| Cancer center/tertiary referral treatment center | 27 | 27.8 |
| Other academic/teaching hospital | 12 | 12.4 |
| Other nonteaching hospital | 11 | 11.3 |
| Private hospital or clinic | 47 | 48.5 |
| Geographic location |  |  |
| Northeast | 26 | 26.8 |
| Midwest | 21 | 21.7 |
| South | 30 | 30.9 |
| West | 20 | 20.6 |
